# Catheter‐associated bladder mucosal trauma during intermittent voiding: An experimental study in pigs

**DOI:** 10.1002/bco2.295

**Published:** 2023-11-30

**Authors:** Kristian Stærk, Brit Schrøder, Louise Kruse Jensen, Troels Petersen, Thomas Emil Andersen, Lene Feldskov Nielsen

**Affiliations:** ^1^ Department of Clinical Research University of Southern Denmark Odense Denmark; ^2^ Department of Clinical Microbiology Odense University Hospital Odense Denmark; ^3^ Preclinical, R&D, Innovation Coloplast A/S Humlebaek Denmark; ^4^ Pathobiological Sciences University of Copenhagen Copenhagen Denmark

**Keywords:** bladder trauma, intermittent catheter, large animal model, microhole zone catheter, mucosal suction, pig, urinary catheter

## Abstract

**Objective:**

The objective of this study is to characterize bladder mucosal trauma associated with intermittent catheterization with conventional eyelet catheters (CECs) and to assess if a microhole zone catheter (MHZC) design concept reduces this adverse effect.

**Materials and Methods:**

A porcine model was developed to reflect human catheterization and bladder drainage. Nine pigs were randomized for catheterization with CEC (*n* = 6) or MHZC (*n* = 3). The bladder was drained repeatedly 20 times through the catheter. Cystoscopy was performed before and after the procedure, and bladders were analysed by histopathology. Two additional pigs were used for cystoscopy visualization of suction events in vivo. Cystoscopy, gross pathology, histopathological score, leucocyte infiltration, and intracatheter pressure at flow stops during voiding were compared for each group.

**Results:**

A significant higher pressure gradient was measured inside the CECs compared with MHZCs during flow stop. Consequently, CECs resulted in suction events inflicting bladder trauma characterized by loss of epithelium, oedema, haemorrhage, and neutrophil tissue infiltration. No significant trauma was identified when using MHZC.

**Conclusions:**

Considerable mucosal bladder trauma is inflicted by CECs which may be an overlooked risk factor for urinary tract infection. Catheters can be designed to minimize mucosal suction and reduce associated trauma. This may be a solution to reduce infection frequency and increase user comfort. Furthermore, the study demonstrates the potential of pigs as an attractive animal model for investigating urinary catheter performances.

## 1 INTRODUCTION

Urinary catheters are one of the most frequently deployed medical devices and a well‐established method to facilitate voiding in patients who have lost the ability for controlled micturition.^1^ Regrettably, catheterization of the urinary tract is a major risk factor of catheter‐associated urinary tract infections being the main healthcare associated infections in western countries.^2^ The risk associated with indwelling catheters is largely explained by the continuant presence of a foreign body in the urinary tract supporting the growth of microbial biofilms that can propagate along the catheter and lead to an infection.^3^ However, the pathobiological risk factors associated with intermittent catheterization (IC), where the catheter is removed immediately after drainage, are less clear. Voiding by IC often ends in a flow stop where residual urine is still present in the bladder, forcing the user to reposition the catheter to reinitiate the flow. The reposition typically needs to be repeated several times to ensure complete bladder emptying. Ex vivo studies have demonstrated that flow stops are associated with the occurrence of a hydrodynamically generated negative pressure gradient with peak pressures at the site of the catheter eyelets that facilitates transient suction events of the urothelium.^4^, ^5^, ^6^ The resulting mucosal trauma from these suction events compromises the urothelial barrier which may be an overlooked risk factor of urinary tract infection (UTI) in patients using IC or indwelling catheters.^7^ Catheter‐associated suction events have been reported in humans.^5^ However, detailed investigation, that is, pathological and histochemical analysis of catheter‐inflicted bladder trauma, have not been feasible to perform in humans of ethical reasons and not in vivo due to lack of appropriate animal models. Hence, there is currently a significant gap in the literature regarding the mucosal trauma associated with catheter suction events and their potential implication on UTI.^5^

Pigs are preferred models in urologic research due to shared anatomy and physiology and their size supports the use of human‐relevant catheters.^8^, ^9^, ^10^ In this study, we developed an in vivo porcine model suitable for testing catheter‐associated bladder trauma and characterized these traumatic lesions based on mucosal changes (visualized by cystoscopy), gross pathology, and histopathological analysis. Furthermore, we evaluated a novel microhole zone technology intermittent catheter (MHZC) that has been designed to reduce suction events and thus promoting bladder emptying in an uninterrupted urine flow.

## 2 MATERIALS AND METHODS

### 2.1 Animals

Eleven female pigs (Landrace × Yorkshire) were sourced from a Specific‐Pathogen‐Free supplier (Kokkenborg, Denmark). The mean weight of the animals was 37.1 (*SD*: 2.2) kg when the experiment began. The study was conducted in accordance with the ARRIVE guidelines and approved by the Danish Animal Experiments Inspectorate, licence number: 2021‐15‐0201‐00931.

Pigs were premedicated with an intramuscular injection of medetomidine 0.03 mg·kg^−1^, midazolam 0.25 mg·kg^−1^, ketamine 5 mg·kg^−1^, and butorphanol 0.2 mg·kg^−1^. General anaesthesia was induced and maintained with propofol 10 mg mg·kg^−1^·h^−1^ and fentanyl 20 μg·kg^−1^·h^−1^ while achieving normoventilation mechanically. In general anaesthesia, non‐invasive blood pressure, electrocardiogram, heart rate, oxygen saturation, and capnography were monitored. The experiments were nonsurviving, and the animals were euthanized with 5 mL intravenous pentobarbital (200 mg·mL^−1^).

### 2.2 Catheters and intraluminal catheter pressure

Six pigs were catheterized with Charrière 12 conventional eyelet catheters (CECs) from three manufacturers: SpeediCath® (Coloplast, *n* = 2); Vapro™ (Hollister, *n* = 2); and LoFric® (Wellspect, *n* = 2). The CECs were compared with a novel catheter (also Charrière 12) based on a microhole zone technology (Luja™, Coloplast, *n* = 3). The MHZC features >80 microholes (Ø = 0.4 mm) placed in a 6 cm drainage zone (Figure S1). To assess the pressure gradient inside the catheter during voiding, a fibreoptic pressure sensor (FISO‐LS fibre optic pressure catheter, model 75‐0706, Fiso Technology Inc, Canada, with an Evo Chassis to power the sensor and constitute the digital interface for data transfer) was mounted inside the catheter just below the eyelet.

### 2.3 In vivo imaging of catheter voiding

In two pigs, a cystoscope (C‐view, Storz) was inserted through a suprapubic access to visualize the mucosal suction events during transurethral drainage. The procedure was repeated by instilling 100 mL saline to observe multiple catheter‐mucosal interaction.

### 2.4 Catheter‐associated mucosal trauma model

Anaesthetized pigs were placed in supine recumbency. Baseline images of the bladder mucosa were recorded using a cystoscope (27 002 K, Karl Storz). The bladder was emptied through the working channel of the cystoscope until approximately 50 mL was left in the bladder (estimated by the operator). A small fraction was used for dipstick analysis (see below). Hereafter, 180 mL saline was instilled to distend the bladder and allow clear visualization of the mucosa. One hour after cystoscopy, the bladders were catheterized, urine was collected for dipstick analysis, and the remaining urine was completely emptied. For the remaining of the experiment, the catheter was held in place by one person to avoid dislodgement and repositioning of the catheter. Hereafter, 120 mL saline was instilled, and the bladder subsequently drained through the catheter while the pressure changes were recorded (described below). At flow stops, the catheter was turned 360° to dislodge potential mucosa from the eyelets, reflecting the manipulation of catheters that IC users are instructed to perform to facilitate complete emptying. When the full volume (i.e., 120 mL) had been drained, the procedure was immediately repeated for a total of 20 drainages over approximately 2 h. Hereafter, the catheter was removed, and after 1 h, cystoscopy was performed to visualize potential bladder lesions and to collect a urine sample for a dipstick analysis. Lastly, the pigs were euthanized, and the bladders were removed and opened by a longitudinal incision between the ureters. Three bladder tissue specimens were punched out using a drive punch (Ø = 10 mm) at sites corresponding to the location of the catheter drainage holes. Additional specimens were punched out from the apex of the bladder representing internal control tissue.

### 2.5 Histopathology

Bladder specimens were fixed in 10% neutral‐buffered formalin (Sigma‐Aldrich). Following fixation, each specimen was cut into two parts through macroscopically visible pinpoint lesions if present. Both halves were placed in cassettes with the new cutting surface downwards and processed through graded concentrations of alcohol and embedded in paraffin wax. Tissue sections were cut (4 μm) and stained with haematoxylin and eosin (VWR, DK). Haematoxylin and eosin‐stained sections were evaluated patho‐morphologically with special focus on identifying tissue destruction and the vascular phase of acute inflammation within mucosa and submucosa. The following parameters were registered with a yes or no: epithelial loss, haemorrhage, hyperaemia, and oedema. Registration with a “yes” resulted in one point, and thereby, a total of four points could be given in histopathology score.

### 2.6 Immunohistochemistry (IHC)

IHC based on a primary monoclonal antibody towards MAC387 (Bio‐rad, MCA874G, UK) was used for in situ identification of infiltrating neutrophil granulocytes. The paraffin‐embedded tissue blocks were deparaffinized and cut into sections of 2–3 μm. Tris‐EDTA buffer pH 9 was used for antigen retrieval. After washing in Tris‐buffered saline (TBS) solution (2 × 5 min), the tissue samples were subjected to blocking of endogenous peroxidases in 0.6% H_2_O_2_ for 15–20 min. Afterwards, the samples were subjected to UltraVision Protein Block (AH Diagnostics, DK) for 5 min to prevent additional unspecific staining. Immunostaining was carried out using the UltraVision indirect horse radish peroxidase polymer‐amplification technique. After 30 min of incubation with horse radish peroxidase polymer (LabVision, DK) and subsequent washing in TBS, the slides were incubated with a red chromogen solution (AEC, LabVision, DK) and washed in distilled water. All stains were counterstained with haematoxylin (VWR, DK) and finally mounted in glycerol‐gelatin (VWR, DK). A porcine lymph node was used as positive control, and a monoclonal Mouse, IgG1 antibody (Agilent, X0931, DK) was used as nonsense control. Slides were evaluated for the distribution of positive cells. The total number of Monocyte/Macrophage/Granulocyte antibody (MAC) positive infiltrating neutrophil granulocytes (despite anatomic tissue layer) was counted for each biopsy, that is, counting was done on the slides of two halves and added. A maximum of 100 positive cells were counted. Neutrophils within vessels were not included in the counting. Histopathology and IHC was performed by a blinded pathologist unaware of group allocation and study design.

### 2.7 Dipstick analysis of urine and saline

All urine samples were evaluated using Multistix® 10 SG reagent strip (Siemens), and the results were recorded using an instrumental evaluation (Siemens). Saline collected after every catheterization was tested for presence of blood using the same type of dipstick as for the urine samples.

### 2.8 Statistics

Statistical analyses were performed using Graph Pad Prism version 9.3.1. Comparison between two groups was performed using a nonparametric two‐tailed Mann–Whitney tests. Correlation analysis was performed using nonparametric Spearman correlation.

## 3 RESULTS

### 3.1 Catheter eyelet suction events in vivo

During drainage with CECs, the bladder mucosa was pulled into the eyelets by the negative pressure gradient generated inside the catheter lumen, as recorded by concurrent cystoscopy through a suprapubic access (Figure S2). The force of the suction was strong enough to fix the mucosal invagination in the eyelet which resulted in the tissue being tugged along during repositioning of the catheter (Figure S2C). Using the MHZC, some degree of mucosal suction was also observed in some of the eyelets, but because all eyelets are not closed at the same time during catheterization, the force of the suction appeared too weak to tug the tissue during repositioning of the catheter (Figure S2E and S2F). Video recordings of the mucosal suction are demonstrated in Video S1.

### 3.2 Intraluminal catheter pressure

**FIGURE 1:**
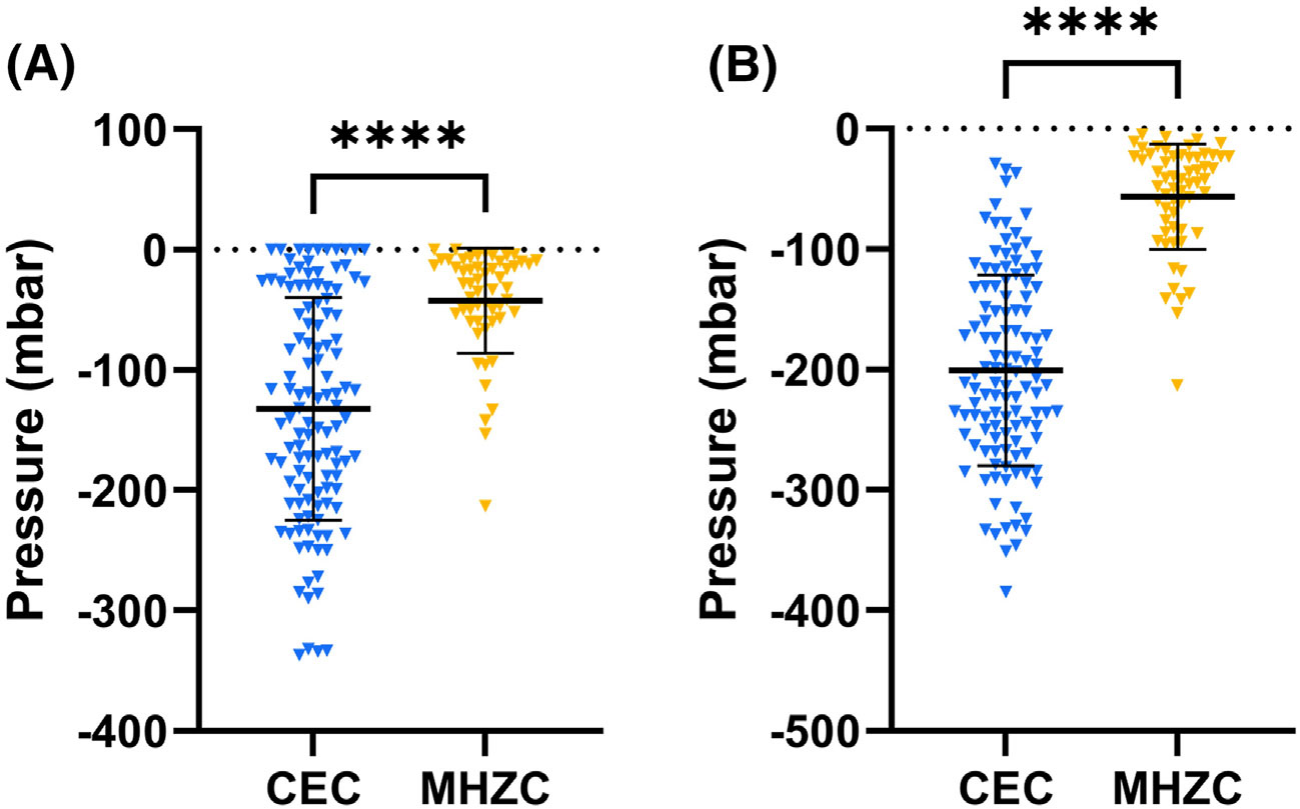
Intraluminal pressure measured inside the catheter at first flow stop (A) and as the highest pressure peak within each drainage (B). A lower negative pressure means a larger pressure impact for the respective catheter type. CEC, conventional eyelet catheter; MHZC, microhole zone catheter. ****P < 0.0001.

The intraluminal pressure inside the catheters was measured during the entire catheterization procedure making it possible to estimate the intraluminal pressure at flow stop until the bladder was completely emptied. For CECs, the average intraluminal pressure at first flow stop was −132.2 mbar ± 7.4 mbar (Figure 1A). Repositioning of the catheter after first flow stop resulted in additional output in most of the drainages with CECs. For the MHZC, the pressure peak at the first flow stop was −42.3 mbar ± 10.8 mbar (*P* < 00001; Figure 1A), and repositioning of the catheter rarely resulted in additional output. The highest pressure peak for CECs was −200.6 mbar ± 6.4 mbar compared with −56.6 mbar ± 8.0 mbar for the MHZC (*P* < 0.0001; Figure 1B).

### 3.3 Cystoscopy, gross pathology, and urinalysis

**FIGURE 2:**
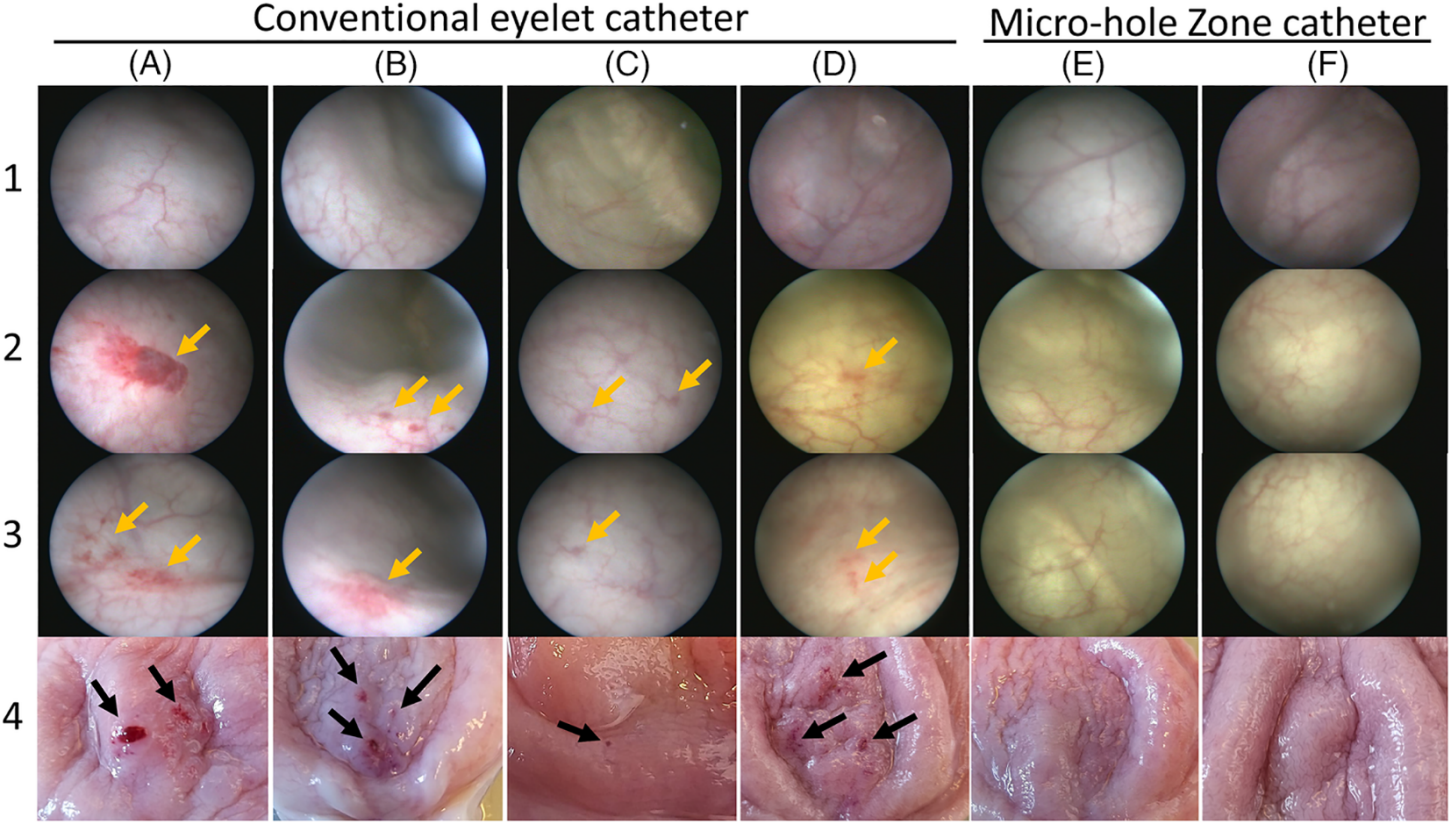
Catheter‐associated mucosal lesions observed by cystoscopy. All bladders appeared normal at baseline (row 1). Significant lesions were observed in the bladder mucosa after the drainage procedures using conventional eyelet catheters, determined by cystoscopy (A–D, rows 2 and 3, yellow arrows) or gross pathology (A–D, row 4, arrows). Lesions were not detected in the bladders from pigs drained by microhole zone catheters (E and F) nor in two of the six pigs drained by conventional eyelet catheters (from two different catheter manufacturers; not shown in the figure).

Based on cystoscopy, all pigs had normal bladder mucosa before catheterization (Figure 2, row 1). After the drainages using CECs, cystoscopy revealed distinct bladder lesions in four of six animals characterized by hyperaemic lesions, haemorrhages, and small round‐shaped changes compatible with the size of the eyelets suggesting that these were formed by suction events (Figure 2). Gross pathology of removed bladders revealed similar lesions congruent with cystoscopy findings (Figure 2). In two animals from the CEC group, no significant mucosal changes were observed during cystoscopy or during gross pathology. Mucosal changes were not observed by cystoscopy or gross pathology in the three pigs drained by MHZCs (Figure 2, columns E and F). Blood (trace amount) was detected in 17 of 119 (14%) drainages with CECs compared with 4 of 56 (7%) for MHZC.

### 3.4 Histopathological findings

**FIGURE 3:**
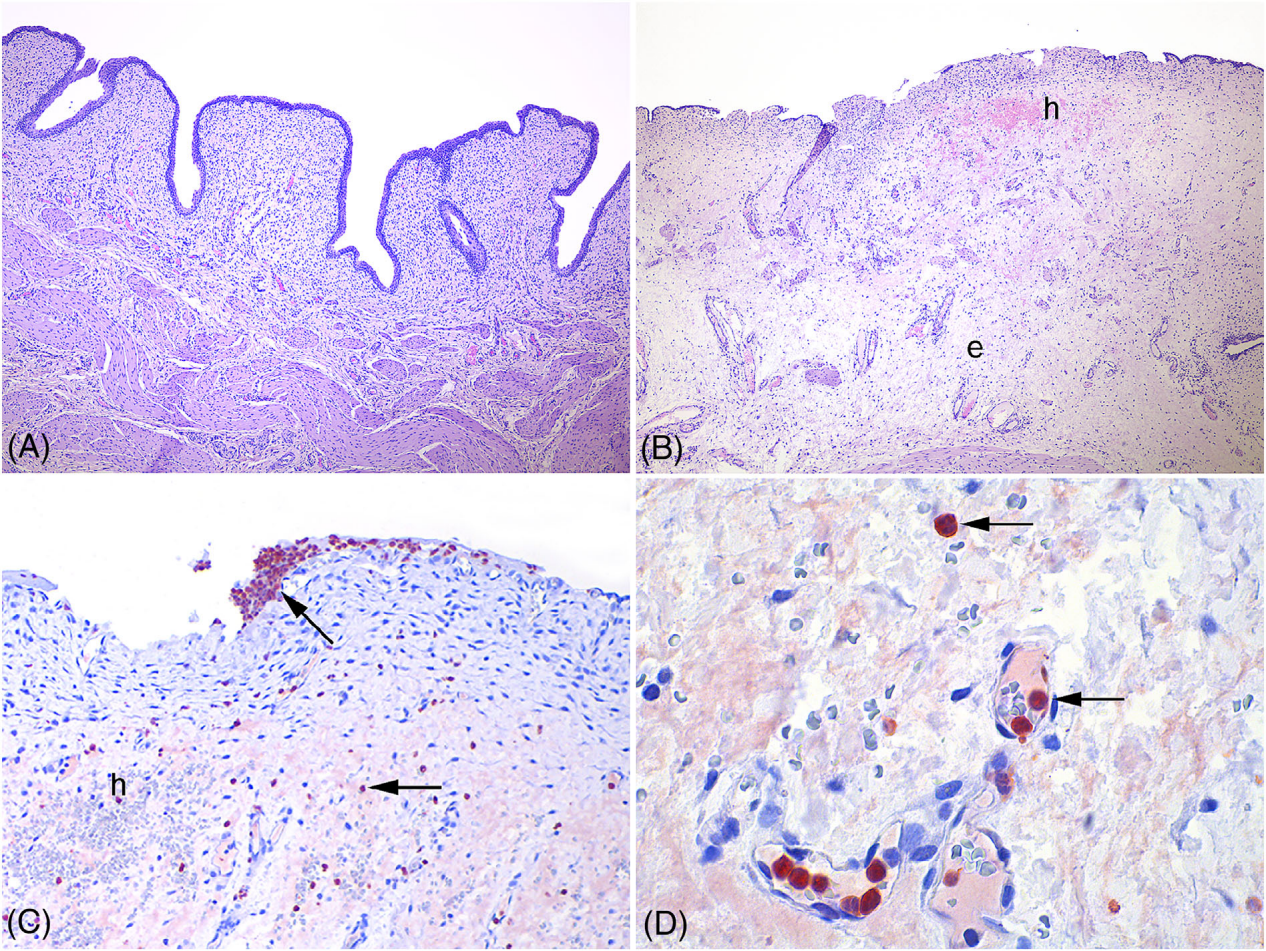
Histology and immunohistochemistry of bladder tissue exposed to repeated catheterizations. (A) Bladder mucosa from a pig catheterized with a microhole zone catheter showing no apparent pathologies. Haematoxylin and eosin stain, 200× magnification. (B) Bladder mucosa from a pig catheterized with conventional eyelet catheter showing loss of bladder epithelia, haemorrhage (h), and massive oedema (e) of the mucosa. Haematoxylin and eosin stain, 200x magnification. (C) Close up of picture (B). Massive neutrophil infiltration (red cells, arrows) is seen across the mucosa. Immunohistochemistry, 400× magnification. (D) Close up of picture (C). Neutrophils within and outside vessels (red cells, arrows). Immunohistochemistry, 600× magnification.

The histology score and neutrophile infiltration counts are summarized in Table 1. CECs were found to significantly increase the histology score (*P* = 0.0003) and the degree of neutrophile infiltration (*P* = 0.0002) in lesion specimens compared with control specimens (from the bladder apex of the same animal). Histological findings in these animals were characterized by loss of epithelia, haemorrhage, and oedema (Figure 3). The bleedings were mainly found in lamina propria beneath the epithelium. No significant difference in histology score or neutrophile infiltration was detected between lesion and control specimens in pigs of the MHZC group (Table 1).

**TABLE 1:** Histopathological outcome.

	Lesion, mean (*SD*)	Control, mean (*SD*)	*P*
Conventional eyelet catheter			
Histology score	2.9 (1.2)	1.2 (0.6)	0.0003
Neutrophile count	46.3 (45.1)	3.3 (3.4)	0.0002
Microhole zone catheter			
Histology score	1.8 (1.0)	0.8 (0.8)	0.097
Neutrophile count	9.9 (11.7)	3.3 (4.9)	0.151

## 4 DISCUSSION

This study found that bladder drainage using CECs induced macroscopic and histopathological lesions characterized by damaged transitional epithelium and inflammation with tissue oedema, haemorrhage, and tissue infiltration of neutrophile granulocytes. Epithelial suction was visualized showing that bladder tissue was sucked into the eyelet holes of CECs. Using a novel catheter type based on microhole zone technology, epithelial suction events were less severe, and macroscopic and histopathological lesions were undetected. The intraluminal catheter pressure showed a significant lower pressure impacting the bladder wall with the MHZC compared with the CEC. Finally, the MHZC facilitated more complete emptying.

To our knowledge, this study is the first of its kind to study bladder trauma associated with IC in vivo. Although the CECs induced bladder trauma of varying severity, macroscopic pinpoint lesions corresponding to the size of the eyelet holes were observed in most bladders, suggesting that this type of lesion is easily inflicted during IC by the CEC design. In a study by Grocela et al., similar pinpoint lesions were observed in human bladders following voiding by a Foley‐type catheter indicating that the present findings from the pig model are translatable to humans.^5^ Because the study by Grocela et al. was performed in human volunteers, no detailed histopathological analysis was performed to characterize the traumatic severity.

Our histopathological analysis of mucosal lesions using CEC revealed distinct signs of early‐stage acute inflammation. This finding convincingly discloses tissue injury due to catheter trauma, which is also supported by the fact that no mucosal lesions were detectable at baseline cystoscopy. The absence of histological lesions in bladder specimens from pigs catheterized with the MHZC is congruent with the finding that peak pressure gradients inside MHZC during drainages were significantly lower (3.5‐fold on average) compared with CECs. Interestingly, peak pressure in CECs was not always associated with the first flow stop, that is, diminishing bladder volumes did not reduce the severity of mucosal suction. Consequently, significant suction events and trauma may occur repetitively during each drainage. The pressure analysis demonstrated that suction events lead to pressure gradients of varying intensity, and during some catheterizations, no pressure gradient was generated at all. It is likely that a certain negative pressure gradient must be reached before significant suction events occur with the capacity of causing trauma. No trauma was detected in two pigs catheterized with CECs, and it is possible that the negative pressure gradient in these animals may have been too low to cause trauma. We know bowel content has influence on the positioning of the bladder and large bowel content often pushes the bladder to the left side. This may influence the contact between the mucosa and the catheter or influence bladder pressure. These anatomical factors could possibly explain why the severity of trauma was different between pigs and in some not detectable at all. This is also supported in the literature in which the abdominal pressure ranges between 15 and 50 cmH_2_O, with the highest pressures in patients standing up compared with patients who are sitting in a chair or in bed.^11^, ^12^

We used pigs, which is the only convenient animal for studying human‐relevant catheter performance.^9^, ^10^, ^13^ Others have used mice, rabbits, and dogs for investigating catheter‐associated urinary tract infection, but these animals are not large enough to accurately recapitulate the human bladder anatomy or to support catheter sizes designed for adults.^13^, ^14^, ^15^ Identical catheter size is critical when analysing mucosal trauma, as the shape and size of eyelets and catheter shaft are shown to influence the hydrodynamic pressure gradient that ultimately dictates mucosal suction and associated trauma.^6^ Arguably, the use of pigs makes the results more translatable to human; however, pigs are more expensive than smaller animal models precluding the use of large sample sizes. A limitation to this study is the experimental approach of 20 repeated drainages which does not truly reflect the use in human patients of roughly five to six drainage per day. As such, the amount of trauma inflicted on the pig's bladder in this setup may be exaggerated compared with human use. However, each bladder lesion observed likely reflects the impact of single suction event, given that two suction events in the exact same place would be improbable. Said bladder lesion could have emerged following the first catheterization as well as the last. Therefore, we argue that the individual lesion is an acceptable approximation to what may happen in humans, and the use of 20 catheterizations mainly increases the likelihood of identifying such catheter‐associated injuries without increasing injury severity overall.

Our study only investigated the immediate trauma and acute inflammation, which may be different from the inflammation following daily, prolonged use. Human IC users are catheterized for long periods, often years, and the cumulative trauma of these ongoing catheterizations is difficult to reproduce experimentally. One reason for this is that the pigs cannot be sedated numerous times daily, due to animal welfare considerations, which would be required for assessing long‐term impact. Nonetheless, the distinct and consistent bladder lesion observed by the described approach demonstrates the usefulness of this experimental model for assessing acute catheter‐inflicted bladder trauma.

Trauma during IC is described as a risk factor for UTI.^15^ Single‐use catheters with hydrophilic coating are shown to reduce urethral trauma and are associated with significantly less UTIs compared with uncoated catheters.^16^ This suggests that catheters which minimize bladder mucosal trauma, such as the MHZC, may reduce the risk of UTI in IC users. More studies are needed to conclude on this potential causality.

## 5 CONCLUSION

Urinary drainage by IC using CECs induces bladder mucosal trauma characterized by loss of transitional epithelium, oedema, and haemorrhage. Reducing hole size and increasing hole numbers as applied in the MHZC reduced the pressure associated with suction events and consequently inflicted significantly less tissue trauma. This suggests that a drawback, and potential risk factor of UTI, associated with traditional ICs can be circumvented by rethinking basic catheter designs. Furthermore, our study demonstrates the pig as an attractive animal model for investigating catheter performances.

## AUTHOR CONTRIBUTIONS

**Kristian Stærk:** Conceptualization; methodology; investigation; project administration; writing—original draft; writing—review and editing. **Brit Schrøder:** Conceptualization; methodology; investigation; writing—review and editing. **Louise Kruse Jensen:** Investigation; writing—review and editing. **Troels Petersen:** Investigation; writing—review and editing. **Thomas Emil Andersen:** Conceptualization; methodology; supervision; Writing—review and editing. **Lene Feldskov Nielsen:** Conceptualization; methodology; investigation; resources; writing—review and editing. All authors approved the final version of the manuscript.

## CONFLICT OF INTEREST STATEMENT

B. S., T. P., and L. N. are employed with Coloplast. K. S. and T. A. collaborate with Coloplast but are not financially dependent on Coloplast nor have they received personal compensation from Coloplast in this or other projects. L. J. has no conflict of interests.

## Supporting information


**Figure S1.** Conventional eyelet catheters feature two large eyelets, whereas the Micro‐hole zone catheter features over 80 micro holes (Ø = 0.4 mm) in a 6‐cm drainage zone. The drainage zone enables placement of micro‐holes in the bottom of the bladder for continuous flow of urine.

**Figure S2.** Catheter eyelets visualized by suprapubic cystoscopy. (A). Bladder drainage by conventional eyelet catheters (SpeediCath, Coloplast) resulted in significant mucosal suction (B, blue arrows), which remained fixed in the eyelet during manipulation of the catheter (C). Some but not all the holes of the Micro‐hole Zone catheter (D, red arrows) became blocked through suction events and with less severity than for conventional eyelet catheters (E, F, yellow arrows).

**Data S1.** Supporting information.

**Video S1.** Supporting information.

## Data Availability

All data are presented in the manuscript.
