# Supplementary figures and images for: Catheter‐associated bladder mucosal trauma during intermittent voiding: An experimental study in pigs
Source: BJUI Compass. 2023 Nov 30;5(2):217–23. doi: 10.1002/bco2.295 (PMC10869658; doi:10.1002/bco2.295)

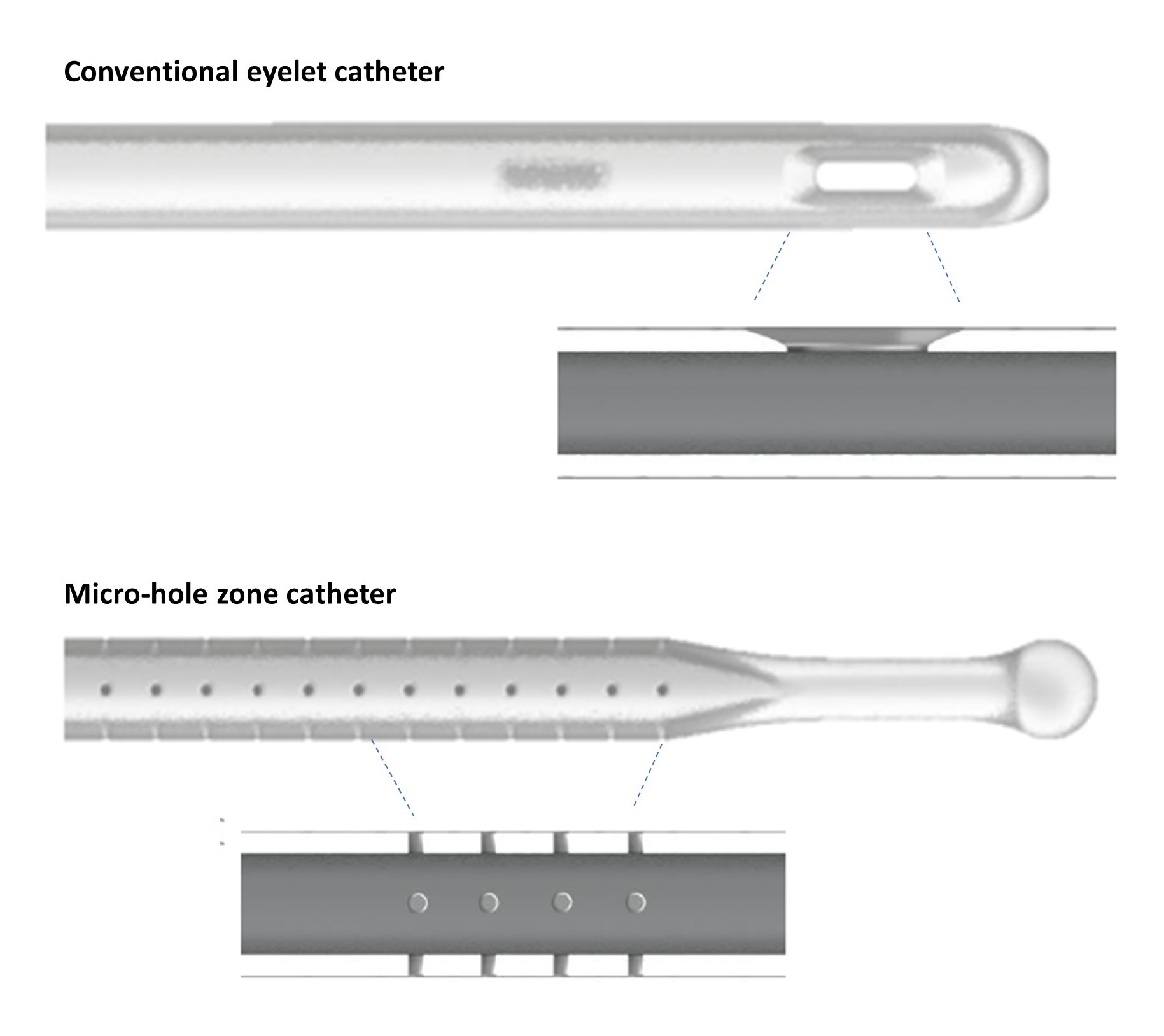

Supplement: Supplementary file 1 — Figure S1. Conventional eyelet catheters feature two large eyelets, whereas the Micro‐hole zone catheter features over 80 micro holes (Ø = 0.4 mm) in a 6‐cm drainage zone. The drainage zone enables placement of micro‐holes in the bottom of the bladder for continuous flow of urine. [file BCO2-5-217-s004.jpg]

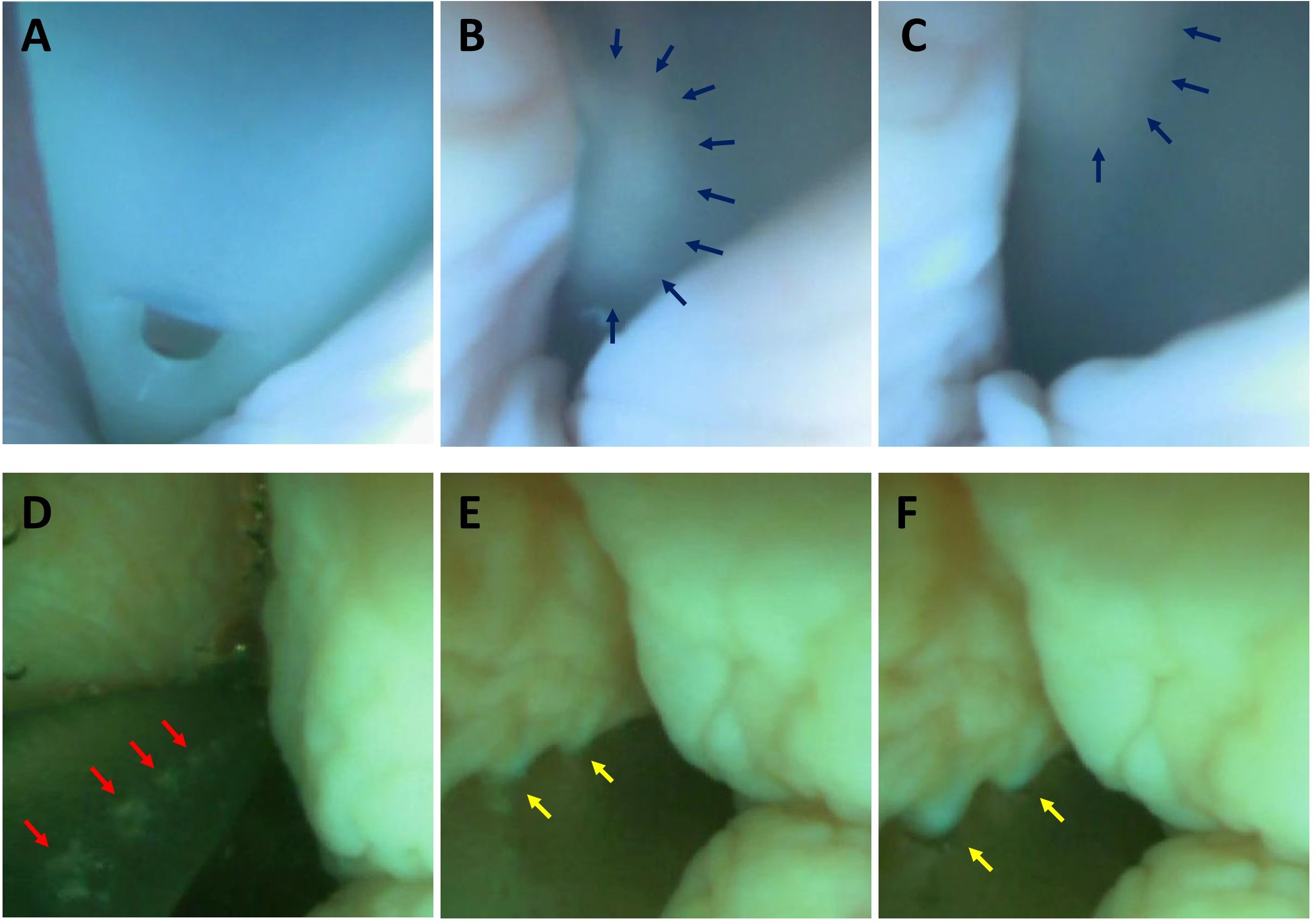

Supplement: Supplementary file 2 — Figure S2. Catheter eyelets visualized by suprapubic cystoscopy. (A). Bladder drainage by conventional eyelet catheters (SpeediCath, Coloplast) resulted in significant mucosal suction (B, blue arrows), which remained fixed in the eyelet during manipulation of the catheter (C). Some but not all the holes of the Micro‐hole Zone catheter (D, red arrows) became blocked through suction events and with less severity than for conventional eyelet catheters (E, F, yellow arrows). [file BCO2-5-217-s002.jpg]
