# Supplementary material for: Catheter‐associated bladder mucosal trauma during intermittent voiding: An experimental study in pigs
Source: BJUI Compass. 2023 Nov 30;5(2):217–23. doi: 10.1002/bco2.295 (PMC10869658; doi:10.1002/bco2.295)
Supplement: Supplementary file 3 — Data S1. Supporting information. [file BCO2-5-217-s001.pdf]

BJU INTERNATIONAL AUTHOR PRE-SUBMISSION CHECKLIST

Please complete this checklist and upload together with your manuscript submission at https://mc.manuscriptcentral.com/bjui

Please note that manuscripts submitted with an incomplete or incorrectly completed form will be returned to the authors without review

GUIDELINES	Yes	No/Not Applicable
**BJU International Author Guidelines**. We have read and adhere to the Author Guidelines	☑	
**BJU International How to write a Manuscript.** We have read BJUI's Advice on preparing a scientific manuscript	☑	☐
**Plagiarism check.** We accept that our manuscript may be screened for plagiarism against previously published work	☑	
**Declaration of Interests.** We include a full statement of declaration of interests for all authors	☑	
**Ethics.** We adhere to Best Practice Guidelines on Publication Ethics	☑	
**References.** References conform to the Vancouver style	☑	
**Funding** (if applicable). We include information regarding research funding	☑	☐
**Reporting of complications** (if applicable)**.** We have used the Clavien-Dindo classification of surgical complications and follow the EAU guideline recommendations (please see Author Guidelines for details)	☐	☑

REPORTING STATISTICS, TABLES AND FIGURES	Yes	No/Not Applicable
**Reporting Statistics**. We have read and adhere to the BJU International Guidelines for Reporting Statistics	☑	☐
We adhere to **Rule 2.1.** We follow existing **Reporting Guidelines** for the type of study we are reporting (e.g., CONSORT for RCTs, PRISMA and AMSTAR for Systematic Reviews, STROBE for observational studies, ReMARK for marker studies). Reporting guidelines and templates for checklists and flow charts can be downloaded from the Equator Web site	☐	☑
**Reporting Figures and Tables**. We have read and adhere to the BJU International Guidelines for Reporting of Figures and Tables	☐	☐

BJU International is a company limited by guarantee, incorporated in England, and is a charity. Registered Office: 3 Junction Mews, London W2 1PN. Company no. 1793312 Charity no. 289273

COVER LETTER	Yes	No/Not Applicable
We include a cover letter	☑	
The cover letter summarizes the key message of the manuscript	☑	
The cover letter confirms that the paper has not been submitted elsewhere	☑	
The cover letter includes any additional pertinent information	☑	☐
**Patient consent form for videos** (if applicable). The corresponding author confirms in the covering letter that a signed release form has been received from each patient videoed authorizing the offline and/or online distribution of this video material	☐	☑

BJU International has 8 different Article Types. Please choose the one that applies to your submission:	Yes
(1) Original Article	☑
(2) Review Article	☐
(3) Comment Article	☐
(4) Research Letter	☐
(5) Step-by-Step Article	☐
(6) Case of the Month (by invitation only)	☐
(7) Editorial (only commissioned by the Editor)	☐
(8) Letter to the Editor	☐
	☐

BJU International is a company limited by guarantee, incorporated in England, and is a charity. Registered Office: 3 Junction Mews, London W2 1PN. Company no. 1793312 Charity no. 289273

**Please fill out the information relevant to your Article Type:**		
**(1) Original Article**		
	**Word count:** max 4000 words (type the actual word count in the box)	
	**Abstract format:** *Objectives; Subjects/patients (or materials) and methods; Results; Conclusion*	☑
	**Key words:** 5–10 key words	☑
	**Manuscript text subheadings:** *Introduction; Subjects/Patients (or Materials) and Methods; Results; Discussion; Acknowledgements; References*	☑
	**Legends to Figures**	☑
	**Figures:** max 3 (type the number of figures in the box)	
	**Tables:** max 3 (type the number of tables in the box)	
	**References:** max 30 (type the number of references in the box)	
**(2) Narrative Review/Systematic Review**		
	**Word count:** max 4000/5000 words (type the word count in the box)	
	**Figures or Tables:** max 6 total (type the number in the box)	
	**References:** 50/no max	☐
	**Systematic Reviews:** The study was pre-registered in PROSPERO	☐
**(3) Comment Article**		
	**Word count:** max 1000 words (type the actual word count in the box)	
	**Figure or Table:** max 1	☐
	**References:** max 6 (type the number of references in the box)	
**(4) Research Letter**		
	**Subheadings.** The manuscript has NO subheadings	☐
	**Word count:** max 1200 words (type the actual word count in the box)	
	**Figure or Table:** max 1	☐
	**References:** max 8 (type the number of references in the box)	
**(5) Step-by-Step Article**		
	**Word count:** max 1200 words (type the actual word count in the box)	
	**Figure or Table:** max 3 for key stages of the procedure	☐
	**References:** max 8 (type the number of references in the box)	
	**Video:** max 8 minutes	☐
**(6) Case of the Month**		
	We have been invited by a BJUI Editor to write this article	☐
**(7) Editorial**		
	We have been invited by a BJUI Editor to write this article	☐
	**Abstract.** The manuscript has NO abstract	☐
	**Word count:** 500–800 words (type the actual word count in the box)	
	**Figure or Table:** max 1 total	☐
	**References:** max 6 (type the number of references in the box)	
**(8) Letter to the Editor**		
	**Word count:** max 500 words (type the actual word count in the box)	☐
	**References:** max 4 (type the number of references in the box)	☐
